# Child Behavior Checklist—Mania Scale (CBCL-MS): Development and Evaluation of a Population-Based Screening Scale for Bipolar Disorder

**DOI:** 10.1371/journal.pone.0069459

**Published:** 2013-08-14

**Authors:** Efstathios Papachristou, Johan Ormel, Albertine J. Oldehinkel, Marinos Kyriakopoulos, María Reinares, Abraham Reichenberg, Sophia Frangou

**Affiliations:** 1 Child Psychiatry Department, Institute of Psychiatry, King's College London, London, United Kingdom; 2 Child and Adolescent Mental Health Services, Maudsley Hospital, London, United Kingdom; 3 Ichan School of Medicine at Mount Sinai, New York City, New York, United States of America; 4 Interdisciplinary Center of Psychpathology and Emotion Regulation, University Medical Center Groningen, University of Groningen, Groningen, The Netherlands; University of Wuerzburg, Germany

## Abstract

**Context:**

Early identification of Bipolar Disorder (BD) remains poor despite the high levels of disability associated with the disorder.

**Objective:**

We developed and evaluated a new DSM orientated scale for the identification of young people at risk for BD based on the Child Behavior Checklist (CBCL) and compared its performance against the CBCL-Pediatric Bipolar Disorder (CBCL-PBD) and the CBCL-Externalizing Scale, the two most widely used scales.

**Methods:**

The new scale, CBCL-Mania Scale (CBCL-MS), comprises 19 CBCL items that directly correspond to operational criteria for mania. We tested the reliability, longitudinal stability and diagnostic accuracy of the CBCL-MS on data from the TRacking Adolescents' Individual Lives Survey (TRAILS), a prospective epidemiological cohort study of 2230 Dutch youths assessed with the CBCL at ages 11, 13 and 16. At age 19 lifetime psychiatric diagnoses were ascertained with the Composite International Diagnostic Interview. We compared the predictive ability of the CBCL-MS against the CBCL-Externalising Scale and the CBCL-PBD in the TRAILS sample.

**Results:**

The CBCL-MS had high internal consistency and satisfactory accuracy (area under the curve = 0.64) in this general population sample. Principal Component Analyses, followed by parallel analyses and confirmatory factor analyses, identified four factors corresponding to distractibility/disinhibition, psychosis, increased libido and disrupted sleep. This factor structure remained stable across all assessment ages. Logistic regression analyses showed that the CBCL-MS had significantly higher predictive ability than both the other scales.

**Conclusions:**

Our data demonstrate that the CBCL-MS is a promising screening instrument for BD. The factor structure of the CBCL-MS showed remarkable temporal stability between late childhood and early adulthood suggesting that it maps on to meaningful developmental dimensions of liability to BD.

## Introduction

Bipolar Disorder (BD) is a complex mental disorder affecting between 0.1% and 4.4% of the general population [1]. BD is the sixth leading cause of disability worldwide particularly amongst adolescents and young adults [2]. This is partly due to the typically early onset of BD with the majority of patients presenting between 19–25 years of age [1], [3]. More important however is the failure in recognizing and treating BD particularly in the early stages of the disorder. The typical delay between onset and diagnosis is 5–10 years [4]–[6] and is associated with greater clinical severity, increased psychosocial morbidity and higher treatment costs [7]–9. Although mania is the diagnostic hallmark of BD [10], [11] the differential diagnosis from Major Depressive Disorder (MDD) is often difficult as BD is commonly dominated by depressive symptoms [12], [13]. Furthermore, BD is also associated with high rates (between 60–80%) of psychotic symptoms during mood episodes [14], [15]. High rates of psychotic symptoms have also been reported in young patients and confirm their role as a key symptom dimension of BD in adolescence [16], [17]. Additional diagnostic challenges arise from the symptomatic overlap between BD and Attention Deficit Hyperactivity Disorder (ADHD), which also presents with poor attentional and emotional regulation [18].

In response to the need for the early identification of individuals at high risk for BD there have been several attempts to develop and validate screening instruments. In adults, one of the most widely studied screening instruments is the Mood Disorder Questionnaire (MDQ) [19], a self-report questionnaire based on the Diagnostic and Statistical Manual of Mental Disorders (DSM) criteria for mania. A positive MDQ screen is based on participants endorsing 7 or more lifetime manic symptoms, several co-occurring, resulting to moderate or serious functional impairment. In outpatient psychiatric settings the MDQ was reported to achieve sensitivity and specificity rates of 67%–83% and 86%, respectively [5]. Although specificity and sensitivity are theoretically independent of prevalence, in practice they are influenced by the clinical composition of the sample (e.g. proportion of severe to mild cases) and interviewers' assumptions about the frequency of a disorder [20]. Typically, in general population samples sensitivity is lower and specificity is higher than that reported in clinical populations. For example, the sensitivity and specificity of the MDQ in the general population are respectively 23–25% and 97–99% [21], [22]. Additionally, many individuals with MDD, anxiety disorders or ADHD screen positive on the MDQ [22], [23].

A significant number of screening instruments for juvenile BD have been developed and have been used mostly in clinical populations. These include the Parent version of the Young Mania Rating Scale (P-YMRS) [24], the Parent General Behavior Inventory (P-GBI) [25], the Adolescent General Behavior Inventory (A-GBI) [26], the Youth Self Report (YSR) [27], the Teacher Report Form (TRF) [28], the Child Mania Rating Scale (CMRS) [29], the Child Behaviour Checklist (CBCL) [30], and the Mood Disorder Questionnaire Adolescent Version (MDQ-A) [31]. The CBCL [30] is the instrument most commonly used to generate profiles relevant to BD in youth. The CBCL is a parent report checklist of 118 items mapping onto multiple aspects of psychopathology over a 6-month period [30], [32]. The CBCL items are grouped in eight behavioural domains: aggressive behaviour, anxiety/depression, attention problems, rule-breaking behavior, withdrawal/depression, somatic complaints, social problems and thought problems [30]. Different scales have been generated based on varied combinations of these behavioural domains. Of relevance to BD, are the Externalizing Scale (comprising item scores from the rule-breaking and aggressive behavior domains) and the CBCL-Pediatric Bipolar Disorder scale (CBCL-PBD) (comprising item scores from the aggressive behavior, anxiety/depression and attention problems domains) [33]. The CBCL-PBD is also referred to as the Dysregulation Profile as it has been associated with disorder involving extensive behavioural and emotional dysregulation [34] including BD [35]. However all available instruments have limited specificity for BD as they have been associated with MDD, ADHD and anxiety disorders [36]–[40].

Therefore there is still a need for screening instruments for BD particularly for use in non-clinical populations of young individuals. In an attempt to address this need we developed and evaluated a new screening scale for BD in children and adolescents based on the CBCL 6–18 [30]. Despite the limited success of previous CBCL-based screening instruments for juvenile BD we decided to use it as the base of the new scale because of its cross-cultural generalizability [41]. However, instead of using summary scores of the existing behavioural domains we constructed this new scale following the methodology defined for DSM-oriented subscale development by Achenbach et al. (2003) [42]. Content validity of the new scale was evaluated by an expert panel of child and adolescent psychiatrists who selected 19 CBCL items that relate directly to the diagnostic criteria for mania as currently operationalized in the DSM5 (details in File S1). The new scale called CBCL-Mania Scale (CBCL-MS) was tested for its psychometric properties, sensitivity and specificity on data from the TRacking Adolescents' Individual Lives Survey (TRAILS) [http://www.trails.nl/en/] [43]. TRAILS is a prospective study of an epidemiologically representative cohort of 2230 Dutch adolescents who were assessed with the full CBCL at age 11,13 and 16. Clinical outcomes were evaluated at age 19 using the Composite International Diagnostic Interview (CIDI) [44]. We also compared the performance of the CBCL-MS against the CBCL-Externalising Scale and the CBCL-PBD to test whether it presents an improvement in terms of accuracy and predictive ability.

## Methods

### Participants

The sample consisted of participants of 2230 Dutch youth participating in the TRacking Adolescents' Individual Lives Survey (TRAILS). The sampling procedure and cohort details for TRAILS have been previously described in detail [43] and can be found at the study website [http://www.trails.nl/en/]. Briefly, the cohort includes children born between 1 October 1989 and 30 September 1991 in a well-defined geographic area in the north Netherlands (information about the representativeness of the sample in S2). Permission to use anonymised data from the TRAILS was granted by the study management committee and ethical approval was granted by the Dutch Central Committee on Research Involving Human Subjects (CCMO). All data were anonymised by a research company TNS NIPO [http://www.tns-nipo.com/].

### Assessments

When TRAILS cohort members were 11, 13 and 16 years old, their parents or parent surrogates completed the CBCL 6–18. Each CBCL item was scored on a three point scale (0 = not true, 1 = somewhat or sometimes true, 2 = very true or often true) on the basis of the preceding 6 months. At age 19 years the diagnostic status of the TRAILS participants was ascertained using the Computer Assisted Personal Interview version 20 (CAPI) of the CIDI [http://www.hcp.med.harvard.edu/wmhcidi/]. The CIDI is a comprehensive, structured interview which was used by trained lay interviewers to assess mental disorders according to current diagnostic systems. It has high test-retest reliability for the diagnosis of BD type I (BD-I) [45] as well as excellent concordance rates with the Structured Clinical Interview for DSM-IV (SCID) for lifetime bipolar spectrum disorders [46]. Diagnostic assessments were conducted blind to participants' CBCL scores.

### Child Behavior Check List - Mania Scale (CBCL-MS)

An expert panel of child and adult psychiatrists, based at the Institute of Psychiatry and the South London and Maudsley NHS Foundation Trust, independently screened all CBCL items to select those that correspond to the DSM operational criteria for mania. As the diagnostic criteria for mania in DSM5 and ICD-10 are identical [www.who.int/classificatios/icd/en/GRNBOOK.pdf] this selection is applicable to both diagnostic systems. In addition, the panel considered CBCL items relating to psychotic-like experiences as childhood and adolescent psychosis and high CBCL total scores are frequently associated with later development of mania [37]–[40]. Following consensus meetings, 19 items were selected for inclusion in the new CBCL- Mania Scale (CBCL-MS) (Table 1). Detailed information on the item selection procedure is included in File S1. The scoring of the CBCL-MS at each assessment age was based on summing the scores of each of the 19 individual items. Scores were then standardized (T scores) following the scoring procedure recommended by Achenbach and Rescorla (2001) [32] using the TRAILS data as the standardization sample. Standardization of the CBCL scores for the CBCL-MS, as well as for other CBCL-based syndrome scales, was performed separately at each assessment age. The CBCL-MS and its scoring are available in File S5.

**Table 1:** Child Behavior Checklist-Mania Scale items and corresponding core and extended DSM-IV criteria for Mania.

CBCL Items	DSM-IV criteria for Mania
**Core Symptoms**	
**37.** Gets in many fights	A distinct period of abnormally and persistently elevated, expansive or irritable mood
**87.** Sudden changes in mood or feelings	
**96.** Thinks about sex too much	
**74.** Showing off or clowning	Inflated self-esteem or grandiosity
**94.** Teases a lot	
**76.** Sleeps less than most kids	Decreased need for sleep (e.g., feels rested after only 3 hours of sleep)
**100.** Trouble sleeping	
**93.** Talks too much	More talkative than usual or pressure to keep talking
**104.** Unusually loud	Flight of ideas or subjective experience that thoughts are racing
**78.** Inattentive or easily distracted	Distractibility (i.e., attention too easily drawn to unimportant or irrelevant external stimuli)
**10.** Can't sit still, restless or hyperactive	Increase in goal-directed activity (at work, at school, or sexually) or psychomotor agitation
**60.** Plays with own sex parts too much	
**41.** Impulsive or acts without thinking	Excessive involvement in pleasurable activities that have a high potential for painful consequences (e.g., engaging in unrestrained buying sprees, sexual indiscretions, or foolish business investments)
**59.** Plays with own sex parts in public	
**Extended Symptoms**	
**34.** Feels others are out to get him/her	Delusions
**85.** Strange ideas	
**89.**Suspicious	
**40.** Hears sound or voices that aren't there	Hallucinations
**70.** Sees things that aren't there	

### Statistical Analysis

Analyses were performed using IBM SPSS Statistics, Version 19 (www.spss.com) and MPlus 6.0 (www.statmodel.com).

#### Reliability and validity of the CBCL-MS

As the CBCL-MS is a new scale the consistency of its items at each assessment wave was evaluated using Cronbach's alpha. In order to determine the number of factors that best describe the latent factor structure of the CBCL-MS at ages 11, 13 and 16 the following criteria were considered: the shape of the scree plot, parallel analysis using a permutated data approach (number of data sets: 5000; confidence interval 95%) [47], [48], the Kaiser criterion as an upper bound for the number of factors to be retained, and the interpretability of the obtained factor structure. In order to conduct the parallel analysis, principal components analysis (PCA) was performed first, with oblique or varimax rotation (as appropriate). For each assessment age, the model fit of the final solutions was established using Confirmatory Factor Analysis (CFA) and assessed using two fit indices, the Root Mean Square Error of Approximation (RMSEA) (cut-off values less than 0.06 indicate good fit and values as high as 0.08 represent reasonable errors of approximation in the population) and the Confirmatory Fit index (CFI) (values above 0.90–0.95 indicate good fit) [49].

#### Sensitivity and Specificity of the CBCL-MS

Omnibus tests using the standardized T scores of the CBCL-MS, the CBCL-Externalizing Scale and the CBCL-PBD were performed to compare the scores of participants with CIDI diagnoses of BD type I (BD-I) to those of healthy participants and participants with other CIDI diagnoses that are considered relevant to BD as they involve mood abnormalities (anxiety or depression) or inattention and behavioral disruption. We present data on Major Depressive Disorder (MDD), General Anxiety Disorder (GAD), and ADHD as the most pertinent exemplars. Finally, Receiver Operating Characteristics (ROC) curves [50] were used to calculate the diagnostic efficiency of the CBCL-MS, CBCL-PBD and CBCL-Externalizing Scale. A ROC curve illustrates the sensitivity (true positive rate) of different cut-offs on the y axis and the 1-specificity (false positive rate) of the corresponding cut-offs on the x axis. In the ROC analysis, the area under the curve (AUC) statistic provides a summary of test performance. AUC values range from 0 to 1 with higher values denoting greater discriminative power and diagnostic efficiency. The focus of the analysis was on BD-I as the usefulness of a test with poor discriminative ability for core syndromal BD would be questionable. However, we also performed ROC analysis using a more expanded definition of caseness that also included BD type II (BD-II) and hypomania with no major depressive episode.

## Results

### TRAILS participants with BD

At age 19, 56 of the TRAILS participants were diagnosed with BD-I. Thirty-four cases had attracted other psychiatric diagnoses prior to being diagnosed with BD; seventeen had a single previous diagnosis either for Oppositional Defiant Disorder (ODD) (n = 5) or Conduct Disorder (CD) (n = 5) or ADHD (n = 4) or GAD (n = 3). Of the remaining seventeen BD cases, eleven had two prior diagnoses (ADHD/ODD = 2, ADHD/CD = 1, ADHD/GAD = 1, ADHD/MDD = 1, ODD/CD = 3, ODD/GAD = 2, ODD/MDD = 1), five had three prior diagnoses (ODD/CD/GAD = 3, ODD/CD/ADHD = 2) and one had four (ODD/CD/ADHD/GAD).

### Internal consistency and factor structure of the CBCL-MS

**Figure 1:**
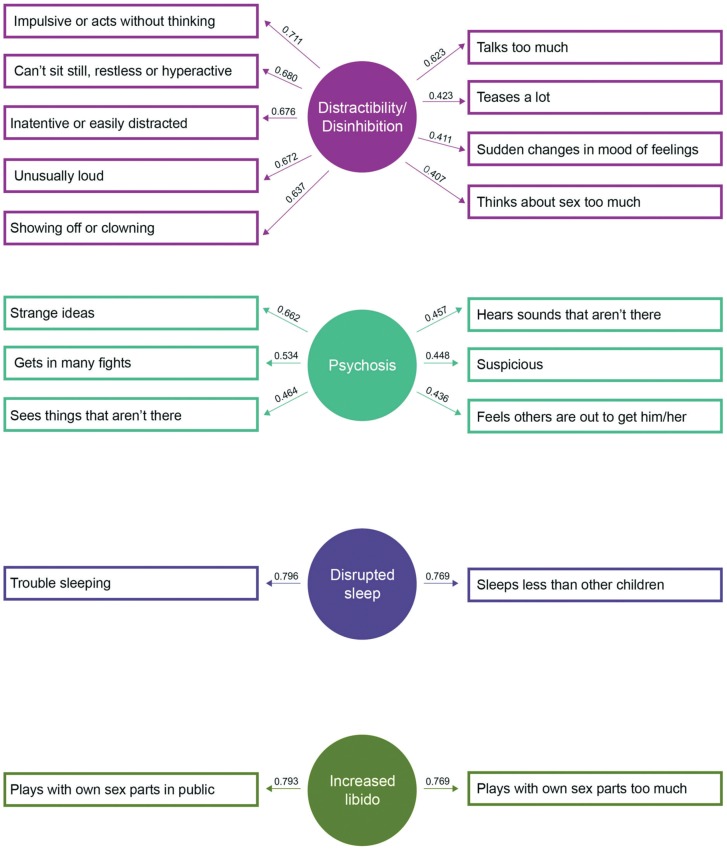
Factors and Factor Loadings of the Child Behavior Checklist-Mania Scale.

Reliability analysis demonstrated high internal consistency for the 19 items of the CBCL-MS at all assessment ages (Cronbach's alpha≥80; total item correlation >0.37). A PCA of the CBCL-MS data assessed at age 16 extracted four factors corresponding to: (1) distractibility/disinhibition (2) psychotic symptoms (3) increased libido (4) disrupted sleep (Figure 1). These factors were plausible and interpretable as items' loading segregated among the four factors as shown in File S3 and Table S2. The factor structure represents the orthogonal solution of the PCA, as the factors extracted using oblique rotation were only weakly correlated (*r*<0.3). Both the Parallel Analysis and Kaiser's criterion supported the retention of four factors. The scree plot of the extracted eigenvalues from the parallel analysis is given in Figure S1, available on line. Analyses of the CBCL-MS data at ages 11 and 13 years resulted in an almost identical factor structure indicating longitudinal stability of this solution (File S3 and Tables S2, S3 and S4). Confirmatory factor analyses further supported the goodness of fit of 4-factor structure (RMSEA≤0.05 and CFI≥0.92).

### Discriminative ability and performance of the CBCL-MS

**Figure 2:**
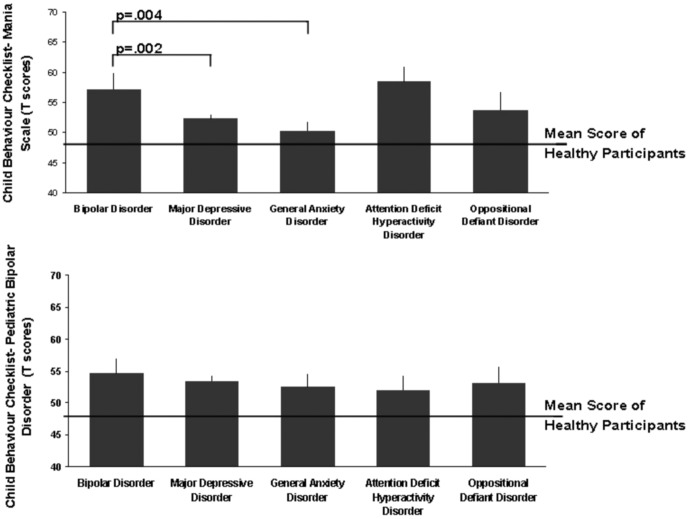
Child Behavior Checklist-Mania Scale Scores in TRAILS Participants.

Table 2 presents the mean total and CBCL-MS factor scores for TRAILS participants who were diagnosed with BD-I and for those who did not have any lifetime psychiatric diagnosis. Participants with BD had significantly higher mean total CBCL-MS scores compared with participants with MDD (n = 178; p = 0.002) and GAD (N = 20; p = 0.004) but not ADHD (N = 26; p>0.05) (Figure 2).

**Table 2:** Child Behavior Checklist-Mania Scale total and Factor Scores in TRAILS participants with Bipolar Disorder (BD) and healthy participants.

	BD participants (N = 56)	Healthy Participants (N = 1201)	
	Mean Score (Standard Deviation)		p
**Total CBCL-MS**	57.28 (16.08)	49.33 (8.95)	<0.001
**Distractibility/Disinhibition**	56.01 (14.71)	49.18 (8.98)	<0.001
**Psychotic Symptoms**	56.19 (20.20)	49.44 (8.84)	<0.001
**Increased Libido**	51.99 (12.28)	49.48 (8.88)	0.088
**Disrupted Sleep**	54.42 (12.28)	49.63 (9.33)	0.002

**Figure 3:**
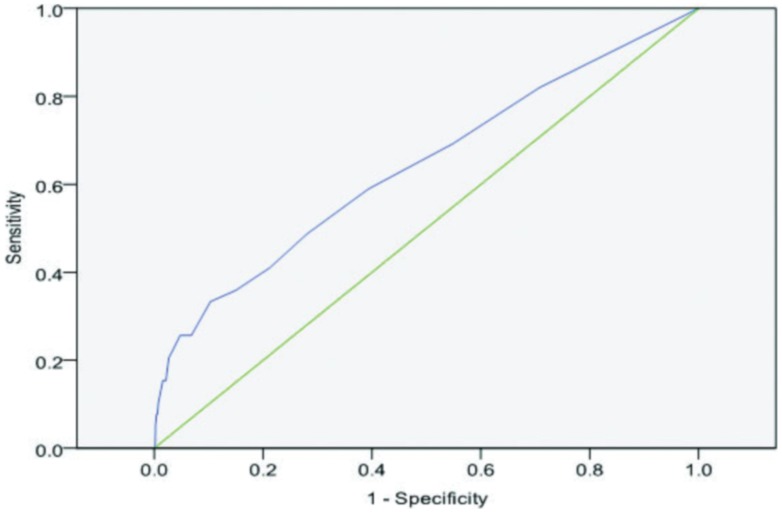
Receiver Operating Characteristics curve of the Child Behavior Checklist-Mania Scale for Bipolar Disorder vs. healthy TRAILS participants.

The ROC curve analysis on the CBCL-MS data at age 16 is illustrated in Figure 3. The AUC was 0.64 (p<0.01) which represents a satisfactory performance for a general population sample with low prior probability of true positives. The AUC remained unchanged when caseness was expanded to include BD-II and hypomania without major depressive episode. Moreover, the total CBCL-MS score performed better than the scores of each individual factors used independently or sequentially (details in supporting information S4 and Tables S5 and S6).

Comparison to CBCL-PBD: TRAILS participants with BD-I had significantly higher CBCL-PBD mean scores (56.51, SD = 15.91) in comparison to healthy participants (49.79, SD = 9.69) but not compared to participants with MDD (p = 0.54), GAD (p = 0.51) or ADHD (p = 0.37). ROC curve analysis showed a moderate ability of the CBCL-PBD in distinguishing BD-I cases from healthy TRAILS participants (AUC = 61%, p = 0.002).

Comparison to the Externalizing scale of the CBCL: Externalizing scale mean scores were significantly higher for TRAILS participants with BD-I (58.49, SD = 18.27) in comparison to healthy participants (48.44, SD = 8.25) and participants with MDD (52.00, SD = 9.80) or GAD (50.52, SD = 7.80), but not compared to participants with ADHD (p = 0.23). ROC analysis showed that the Externalizing scale had AUC = 63%, p = 0.003 when discriminating between TRAILS cases with BD-I and healthy participants.

A forward stepwise logistic regression model showed that the CBCL-MS had significantly increased ability to predict BD-I compared to the CBCL-PBD (Wald χ^2^ = 12.69, p<0.001) and the CBCL-Externalizing Scale (Wald χ^2^ = 3.47, p = 0.05).

## Discussion

We present data on the psychometric properties and discriminative ability of the CBCL-MS, a new DSM based screening scale for BD-I based on the CBCL. We demonstrate that the new scale has excellent psychometric properties; its discriminative ability and accuracy in a general population sample of young people represent an improvement over other commonly used scales particularly CBCL-PBD and the CBCL-Externalizing Scale.

### Prevalence and Characteristics of TRAILS participants with BD

The lifetime prevalence of BD-I in the TRAILS sample was 2.5% which is identical to that reported in a recent epidemiological study of US adolescents [51]. Also consistent with previous literature, nearly 61% of BD-I cases in the TRAILS sample had prior diagnoses associated with disruptive behaviour most commonly ADHD and ODD [18], [50]–[54].

### Factor Structure of the CBCL-MS reveals developmentally meaningful dimensions of liability to BD

The structural model of the CBCL-MS consisted of four factors. These factors correspond to dimensions of distractibility/disinhibition, psychosis, increased libido and disrupted sleep. The factor structure of the CBCL-MS showed remarkable temporal stability between the ages of 11 to 16 which strongly supports the notion that it defines developmentally meaningful dimensions of liability to BD. This report is the first to describe developmental dimensions of liability to BD. All other studies have focused on symptom dimensions during acute manic episodes in patients with established BD [54]–[58]. Nevertheless, there are significant similarities. Cassidy and colleagues identified 5 factors in acute mania of which the “psychomotor pressure”, “psychosis” and “increased hedonic” factors correspond to the distractibility/disinhibition, psychosis and increased libido factors in this study [55]. Picardi et al [56] defined a four factor structure of acute mania based on the Brief Psychiatric Rating Scale. The factors they named “mania” and “disorganisation” include items similar to the distractibility/disinhibition factor identified here. In addition their “positive symptoms” factor overlaps with the psychosis factor in this study. Cassano et al [57] identified 5 factors in acute mania of which “psychomotor agitation” and “psychoticism” correspond to the factors of distractibility/disinhibition and psychosis in the TRAILS cohort. All three studies also defined factors relating to dysphoric/euphoric mood and aggression that seem to be present only during acute mania and may not represent an independent dimension of developmental liability to BD.

### Discriminative ability of the CBCL-MS

The overall accuracy was 0.64 for the CBCL-MS and CBCL-Externalising Scale and 0.61 for the CBCL-PBD. The results of the logistic regression comparing the three scales showed that the CBCL-MS was statistically better in predicting BD outcome.

As seen by the ROC, different cut-off scores will influence the sensitivity and specificity of the CBCL-MS. In general population screening the emphasis is usually on specificity thus selecting individuals at highest risk for detailed follow-up assessments. To illustrate this point, in a hypothetical community sample of 10000 youth with a 2.5% prevalence of BD we would expect 250 individuals to have BD (true positives). A CBCL-MS score of 70 or above will correctly identify 8,775 individuals (90% of this sample) as not having BD (true negatives). At the same threshold, 1,050 individuals will be classified as possible cases. This sample will include 75 true cases of BD (true positives) and 975 individuals without BD (false positives). At first glance, one might be concerned about the number of false positive cases. However, those scoring above 70 in the CBCL-MS were at a six-fold increased risk for BD (Positive Predictive Value: 16.57%; Negative Predictive Value: 98.01%) compared to the rest of the sample and therefore they represent a high risk group. The field of early intervention in BD is currently in its infancy [59] but as effective therapies become available [60] scales such as the CBCL-MS may contribute to the identification of those at high risk.

None of the scales differentiated participants with BD from those with ADHD in terms of mean scores. The relationship between these two disorders is complex. Available evidence suggests at least partially overlapping aetiology and pathophysiology for BD and ADHD because of familial co-segregation of the two disorders [61], [62], commonalities in their neurobiological correlates [18], [63], [64], and frequent comorbidity [18]. Additionally, there is significant overlap in the symptoms of the two disorders particularly with regards to increased activity, talkativeness and mood dysregulation [10], [11]. Two main features distinguishing BD from ADHD have been proposed. Geller and colleagues emphasized the importance of either elevated mood or grandiosity for a diagnosis of mania [65]. However, in our study these symptoms clustered with others in one factor and did not differ across the two diagnostic categories. Others have suggested that episodicity is more indicative of BD than ADHD [66] but this distinction seems less clear in children and adolescents [51]. It is therefore possible that scales based on observed behaviour lack assay sensitivity in distinguishing between BD and ADHD.

### Methodological Issues and Future Directions

The present study has a number of strengths and limitations. The TRAILS sample is representative of the population of young people in the Netherlands (Table S6). The prevalence of BD in the TRAILS is nearly identical to that of general population samples elsewhere [1], [51] which supports the generalizability of findings. Case ascertainment in the TRAILS does not depend on help-seeking behaviour or concern about impairment or severity and thus the sample is free from referral bias present in clinical populations. However, the CIDI although widely used is designed for lay interviewers who rely on its structured format and may not probe or interpret participant responses further. Case ascertainment was conducted at age 19. Since participants have not yet passed the entire period of risk for BD it is possible that further cases of BD may present in the future.

The CBCL-MS performed well within the context of a general population sample, it represents an improvement on available scales and could contribute to future public health initiatives for the identification of youth at high risk for BD. Its accuracy is moderate and in the same broad range of the other CBCL-based screening instruments. Although it could be argued that this is reflects limitations in the CBCL we would suggest that behavioural ratings alone are unlikely to provide us with high levels of accuracy in case identification for BD or any mental disorder. However a great strength of this study was the availability of CBCL assessments at multiple time points from late childhood to early adulthood. This allowed us to test the temporal stability of the CBCL-MS factors which supports the validity of these dimensions as developmentally meaningful premorbid indicators of BD.

## Supporting Information

Figure S1 **Scree Plot.**
(TIF)

File S1 **Selection of Items for the CBCL-MS.**
(DOC)

File S2 **Representativeness of the TRAILS sample.**
(DOC)

File S3 **Psychometric properties of the CBCL-MS across ages.**
(DOC)

File S4 **Discriminative ability of the CBCL-MS.**
(DOC)

File S5 **Appendix: CBCL-MS (scale and scoring sheet).**
(PDF)
