# Supplementary figures and images for: Child Behavior Checklist—Mania Scale (CBCL-MS): Development and Evaluation of a Population-Based Screening Scale for Bipolar Disorder
Source: PLoS One. 2013 Aug 14;8(8):e69459. doi: 10.1371/journal.pone.0069459 (PMC3743889; doi:10.1371/journal.pone.0069459)

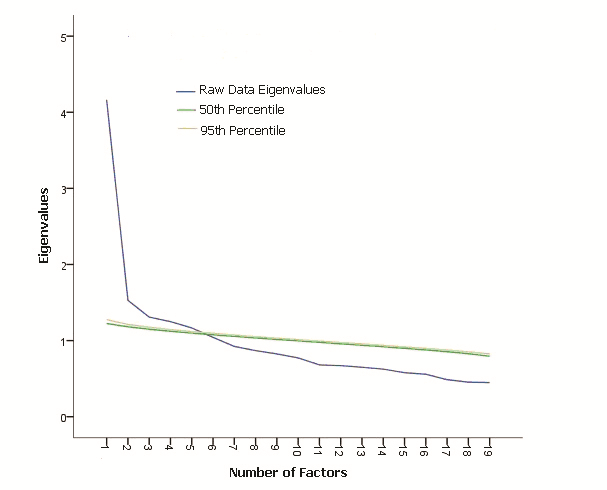

Supplement: Figure S1 — Scree Plot. (TIF) [file pone.0069459.s001.tif]
