# Supplementary material for: Child Behavior Checklist—Mania Scale (CBCL-MS): Development and Evaluation of a Population-Based Screening Scale for Bipolar Disorder
Source: PLoS One. 2013 Aug 14;8(8):e69459. doi: 10.1371/journal.pone.0069459 (PMC3743889; doi:10.1371/journal.pone.0069459)
Supplement: File S1 — Selection of Items for the CBCL-MS. (DOC) [file pone.0069459.s002.doc]

**S1 - Selection of Items for the CBCL-MS**

We followed a typical content validity procedure (Grant & Davis, 1997; Lengua et al, 2001) which involves content experts who judge each item of an instrument against the definition of the domains intended to measure and score its relevance on a 4-point scale ranging from 1 (irrelevant) to 4 (highly relevant). In our study, content validity was assessed by a panel of 10 qualified child and adolescent psychiatrists (average number of years of clinical and research experience: 8; range: 4—20 years). These experts were representative of the professional settings (inpatient, outpatient, academic) within the South London and Maudsley NHS Foundation Trust. They rated the relevance of each item from the total item pool of the CBCL 6-18 on a scale of 1-4 against each of the symptom domains of mania as defined in the DSM-IV. The content validity index (CVI) for each item was established by calculating the proportion of experts scored it as relevant (score 3) or highly relevant (score 4) for any of the domains. A minimum item CVI of 0.80 (Grant & Davis, 1997) was required for inclusion in the second stage where final item inclusion to the CBCL-MS was based on unanimous agreement.

References

Grant JS, Davis LL (1997) Selection and use of content experts for instrument development. Res Nurs Health 20:269-274.

Lengua LJ, Sadowski CA, Friedrich WN, Fisher J (2001) Rationally and empirically derived dimensions of children's symptomatology: Expert ratings and confirmatory factor analyses of the CBCL. J Consult Clin Psych 69: 683-698.
