# Supplementary material for: Child Behavior Checklist—Mania Scale (CBCL-MS): Development and Evaluation of a Population-Based Screening Scale for Bipolar Disorder
Source: PLoS One. 2013 Aug 14;8(8):e69459. doi: 10.1371/journal.pone.0069459 (PMC3743889; doi:10.1371/journal.pone.0069459)
Supplement: File S2 — Representativeness of the TRAILS sample. (DOC) [file pone.0069459.s003.doc]

**S2 - Representativeness of the TRAILS sample**

The TRAILS cohort was identified through birth and school registers in the 5 northern municipalities of Holland. Recruitment was based on entire schools participating; 91% of all the schools in the municipalities agreed to take part. Table S1 shows that key variables regarding representativeness of the TRAILS cohort are comparable to those from the 2001 Dutch national census.

| **Table S1. *Characteristics of TRAILS participants at enrolment compared to the 2001 national census data available online from the Centraal Bureau voor de Statistiek (www.cbs.nl)*** | | |
| --- | --- | --- |
|  | **TRAILS** | **2001 Dutch Census** |
| **% Girls** | 50.8% | 48.8%^1^ |
| **% White European** | 89.4% | 93.2%^1^ |
| **Lower parental education^2^** | 32.6% | 30.6% |
| **% children living with both parents^3^** | 84.5% | 84% |
| **Median disposable household income** | 21780 Euros | 20700 Euros |
| *^1^ The census reported on the 10-15 years age range; ^2^Defined as having completed up to lower half of secondary school ^2^ includes married and cohabiting couples* | | |
