# Supplementary material for: Child Behavior Checklist—Mania Scale (CBCL-MS): Development and Evaluation of a Population-Based Screening Scale for Bipolar Disorder
Source: PLoS One. 2013 Aug 14;8(8):e69459. doi: 10.1371/journal.pone.0069459 (PMC3743889; doi:10.1371/journal.pone.0069459)
Supplement: File S3 — Psychometric properties of the CBCL-MS across ages. (DOC) [file pone.0069459.s004.doc]

**S3 - Psychometric properties of CBCL-MS across ages**

Reliability analyses demonstrated high internal consistency for the 19 items of the CBCL-MS at all assessment ages (Cronbach’s alpha≥0.80; item total correlation >0.37). PCA analyses of the CBCL-MS data at ages 11, 13 and 16 years resulted in an almost identical factor structure and explained 48.18%, 46.06% and 44.94% of the variance of the items respectively. The four factors corresponded to: (1) distractibility/ disinhibition (2) psychotic symptoms (3) increased libido (4) disrupted sleep. Items’ loading for the four factors are shown in Tables S2-S4. Parallel analyses and Kaiser’s criterion both supported the retention of four factors. The scree plot of the extracted eigenvalues from the parallel analysis at age 16 is given in Figure S1.

Fit indices of Confirmatory Factor Analyses (CFA) established good fit of the data [Root Mean Square Error of Approximation (RMSEA) values were 0.04, 0.04 and 0.05, and Confirmatory Fit index (CFI) values were 0.97, 0.96 and 0.92 for the three assessment points respectively].

**Table S2**. Items, factors and item loadings of the CBCL-MS at age 16

| **CBCL Items** | **Distractibility/ Disinhibition** | **Psychotic Symptoms** | **Disrupted Sleep** | **Increased Libido** |
| --- | --- | --- | --- | --- |
| **41.** Impulsive or acts without thinking | **.711** | .109 | .043 | .025 |
| **10.** Can’t sit still, restless or hyperactive | **.680** | .053 | .081 | .108 |
| **78.** Inattentive or easily distracted | **.676** | .002 | .117 | .047 |
| **104.** Unusually loud | **.672** | .177 | .038 | .073 |
| **74.** Showing off or clowning | **.637** | .092 | .018 | .070 |
| **93.** Talks too much | **.623** | .007 | .103 | -.047 |
| **94.** Teases a lot | **.423** | .390 | -.145 | .159 |
| **87.** Sudden changes in mood or feelings | **.411** | .299 | .237 | -.093 |
| **96.** Thinks about sex too much | **.407** | -.026 | .201 | .001 |
| **85.** Strange ideas | .028 | **.662** | .301 | -.101 |
| **37.** Gets in many fights | .290 | **.534** | -.131 | .225 |
| **70.** Sees things that aren’t there | -.027 | **.464** | .326 | -.054 |
| **40.** Hears sound or voices that aren’t there | -.074 | **.457** | -.054 | .137 |
| **89.**Suspicious | **.406** | **.448** | .150 | -.169 |
| **34.** Feels others are out to get him/her | .384 | **.436** | .055 | -.108 |
| **100.** Trouble sleeping | .162 | .127 | **.796** | .030 |
| **76.** Sleep/s less than most kids | .209 | .038 | **.769** | .097 |
| **59.** Plays with own sex parts in public | .029 | .109 | .055 | **.793** |
| **60.** Plays with own sex parts too much | .086 | -.043 | .038 | **.749** |
| **Item numbered as in the CBCL (6/18)** | | | | |

**Table S3**. Items, factors and item loadings of the CBCL-MS at age 13

| **CBCL Items** | **Distractibility/ Disinhibition** | **Psychotic Symptoms** | **Increased Libido** | **Disrupted Sleep** |
| --- | --- | --- | --- | --- |
| **41.** Impulsive or acts without thinking | **.704** | .055 | .014 | .061 |
| **104.** Unusually loud | **.696** | -.022 | .024 | .108 |
| **10.** Can’t sit still, restless or hyperactive | **.695** | -.041 | .060 | .052 |
| **78.** Inattentive or easily distracted | **.680** | .068 | .050 | .080 |
| **74.** Showing off or clowning | **.671** | .126 | -.021 | -.029 |
| **93.** Talks too much | **.597** | -.033 | -.035 | .158 |
| **94.** Teases a lot | **.561** | .213 | .120 | -.053 |
| **87.** Sudden changes in mood or feelings | **.489** | .169 | -.013 | .226 |
| **89.**Suspicious | **.406** | .394 | -.087 | .152 |
| **37.** Gets in many fights | **.399** | .273 | .119 | -.122 |
| **96.** Thinks about sex too much | .313 | .176 | .272 | -.048 |
| **70.** Sees things that aren’t there | -.003 | **.789** | -.063 | .025 |
| **40.** Hears sound or voices that aren’t there | -.036 | **.664** | .140 | .056 |
| **34.** Feels others are out to get him/her | .116 | **.497** | .050 | .095 |
| **85.** Strange ideas | .347 | **.371** | -.023 | .107 |
| **60.** Plays with own sex parts too much | -.017 | .076 | **.842** | .045 |
| **59.** Plays with own sex parts in public | .054 | -.029 | **.825** | .015 |
| **76.** Sleeps less than most kids | .097 | .117 | .014 | **.827** |
| **100.** Trouble sleeping | .125 | .115 | .031 | **.803** |
| **Item numbered as in the CBCL (6/18)** | | | | |

**Table S4**. Items, factors and item loadings of the CBCL-MS at age 11

| **CBCL Items** | **Distractibility/ Disinhibition** | **Psychotic Symptoms** | **Disrupted Sleep** | **Increased Libido** |
| --- | --- | --- | --- | --- |
| **104.** Unusually loud | **.750** | .128 | .049 | .055 |
| **74.** Showing off or clowning | **.706** | .140 | .046 | .048 |
| **10.** Can’t sit still, restless or hyperactive | **.681** | .017 | .077 | .055 |
| **78.** Inattentive or easily distracted | **.678** | .076 | .064 | .049 |
| **41.** Impulsive or acts without thinking | **.676** | .138 | .034 | .098 |
| **94.** Teases a lot | **.628** | .078 | .041 | .031 |
| **93.** Talks too much | **.585** | .047 | .072 | .075 |
| **87.** Sudden changes in mood or feelings | **.532** | .306 | .094 | -.086 |
| **37.** Gets in many fights | **.524** | .037 | .001 | .179 |
| **70.** Sees things that aren’t there | .027 | **.706** | .073 | .053 |
| **40.** Hears sound or voices that aren’t there | -.071 | **.685** | .018 | .182 |
| **85.** Strange ideas | .185 | **.539** | .070 | .086 |
| **89.**Suspicious | .329 | **.483** | .100 | -.039 |
| **34.** Feels others are out to get him/her | .392 | **.470** | .031 | -.108 |
| **76.** Sleeps less than most kids | .138 | .067 | **.857** | .030 |
| **100.** Trouble sleeping | .065 | .166 | **.843** | .009 |
| **60.** Plays with own sex parts too much | .082 | .041 | -.034 | **.825** |
| **59.** Plays with own sex parts in public | .003 | .214 | -.050 | **.703** |
| **96.** Thinks about sex too much | .267 | -.050 | .174 | **.476** |
| **Item numbered as in the CBCL (6/18)** | | | | |
