# Supplementary material for: Child Behavior Checklist—Mania Scale (CBCL-MS): Development and Evaluation of a Population-Based Screening Scale for Bipolar Disorder
Source: PLoS One. 2013 Aug 14;8(8):e69459. doi: 10.1371/journal.pone.0069459 (PMC3743889; doi:10.1371/journal.pone.0069459)
Supplement: File S4 — Discriminative ability of the CBCL-MS. (DOC) [file pone.0069459.s005.doc]

**S4 - Discriminative ability of the CBCL-MS**

We examined the discriminative ability of each factor and of the total CBCL-MS score at the cut-off point of 1 standard deviation above their corresponding population mean. The results are shown in Table S4. The total CBCL-MS score had better discriminative ability than each of the individual factor scores.

We then tested whether sequential application of the factors improved predictive ability for BD over that achieved using the composite CBCL-MS alone. We conducted two regression analyses, a stepwise forward logistic regression model using the 4 factors as predictors and a regression model using the total CBCL-MS score as the single predictor. The final model of the stepwise forward logistic regression contained only the distractibility/disinhibition factor suggesting that none of the remaining three factors improved predictive accuracy (all p values of the competing regression models>.05). However, the Nagelkerke R2 for this model was 1.2% less than that obtained from a regression model using the total CBCL-MS score as a single predictor variable. Results of the two regression models are summarized in Table S5. Therefore, the CBCL-MS appears to be better in terms of its predictive accuracy for BD as a single composite scale in comparison to its factors either used individually or in tandem.

**Table S5. *Discriminative ability of the total CBCL-MS and its Factors***

|  | **Area Under the curve (p value)** | **Sensitivity** | **Specificity** |
| --- | --- | --- | --- |
| **CBCL-MS** | 0.64 (0.003) | 0.33 | 0.90 |
| **Distractibility/Disinhibition** | 0.63 (0.004) | 0.25 | 0.87 |
| **Psychosis** | 0.57 (0.097) | 0.17 | 0.94 |
| **Increased Libido** | 0.53 (0.403) | 0.17 | 0.98 |
| **Disrupted Sleep** | 0.56 (0.167) | 0.23 | 0.90 |

**Table S6**. Regression Models

|  | **Predictor Variables** | **B** | **OR** | **95%CI** | **p** | **X^2^** |
| --- | --- | --- | --- | --- | --- | --- |
| **Model 1^a^** | Distractibility/Disinhibition | .05 | 1.05 | 1.03-1.08 | <.001 | 15.19, p<.001 |
|  | Psychotic Symptoms | -- | -- | -- | .07 |  |
|  | Disrupted Sleep | -- | -- | -- | .14 |  |
|  | Increased Libido | -- | -- | -- | .74 |  |
| **Model 2** | CBCL-MS | .06 | 1.06 | 1.03-1.08 | <.001 | 18.92, p<.001 |

*^a^Stepwise Forward Regression Model*

***Table S6.*** Characteristics of TRAILS participants at enrolment compared to the 2001 national census data available online from the Centraal Bureau voor de Statistiek (www.cbs.nl)

|  | **TRAILS** | **2001 Dutch Census** |
| --- | --- | --- |
| **% Girls** | 50.8% | 48.8%^1^ |
| **% White European** | 89.4% | 93.2%^1^ |
| **Lower parental education^2^** | 32.6% | 30.6% |
| **% children living with both parents^3^** | 84.5% | 84% |
| **Median disposable household income** | 21780 Euros | 20700 Euros |

*^1^ The census reported on the 10-15 years age range; ^2^Defined as having completed up to lower half of secondary school ^2^ includes married and cohabiting couples*
