# Supplementary material for: Child Behavior Checklist—Mania Scale (CBCL-MS): Development and Evaluation of a Population-Based Screening Scale for Bipolar Disorder
Source: PLoS One. 2013 Aug 14;8(8):e69459. doi: 10.1371/journal.pone.0069459 (PMC3743889; doi:10.1371/journal.pone.0069459)
Supplement: File S5 — Appendix: CBCL-MS (scale and scoring sheet). (PDF) [file pone.0069459.s006.pdf]

Child Behaviour Checklist – Mania Scale (CBCL-MS)

Below is a list of items the describe children and youths. For each time that describes your child **now or within the past 6 months**, please circle the **2** if the item is **very true or often true** of your child. Circle **1** if the item is **somewhat or sometimes true** of your child. Circle **0** if the item is **not true** of your child. Please answer all items as well as you can, even if some do not seem to apply to your child.

0=not true   1=somewhat or sometimes true   2= very true or often true

**0**	**1**	**2**	**Can't sit still, restless or hyperactive**
**0**	**1**	**2**	**Feels others are out to get him/her**
**0**	**1**	**2**	**Gets in many fights**
**0**	**1**	**2**	**Hears sound or voices that aren't there**
**0**	**1**	**2**	**Impulsive or acts without thinking**
**0**	**1**	**2**	**Plays with own sex parts in public**
**0**	**1**	**2**	**Plays with own sex parts too much**
**0**	**1**	**2**	**Sees things that aren't there**
**0**	**1**	**2**	**Showing off or clowning**
**0**	**1**	**2**	**Sleeps less than most kids**
**0**	**1**	**2**	**Inattentive or easily distracted**
**0**	**1**	**2**	**Strange ideas**
**0**	**1**	**2**	**Sudden changes in mood or feelings**
**0**	**1**	**2**	**Suspicious**
**0**	**1**	**2**	**Talks too much**
**0**	**1**	**2**	**Teases a lot**
**0**	**1**	**2**	**Thinks about sex too much**
**0**	**1**	**2**	**Trouble sleeping**
**0**	**1**	**2**	**Unusually loud**

Items listed correspond in ascending order to items 10, 34,37,40,41,59,60,70,74,76,78,85,87,89,93,94,96,100,104 of the CBCL (6-18) by Achenbach TM, Rescorla LA. Manual for the ASEBA School-Age Forms & Profiles. Research Center for Children, Youth and Families. University of Vermont, Burlington, VT: 2001.

CBCL-MS Scoring Sheet

CBCL-MS Raw Scores

Percentiles	Age11	Age13	Age16	Average T Scores
99	20	16	16	86
98	18	14	13	79
97	16	12	12	75
96	15	11	10	71
95	14	11	10	71
94	14	10	9	69
93	13	10	8	67
90	11	8	7	63
88	10	7	7	61
85	9	7	6	59
80	8	6	5	57
75	7	5	4	54
70	7	4	3	52
65	6	4	3	51
60	5	3	3	50
55	5	3	2	49
50	4	2	2	47
45	3	2	2	46
40	3	2	1	45
30 - 35	2	1	1	44
25	1	1	0	42
15 - 20	1	0	0	41
0 - 10	0	0	0	40

Average score for healthy individuals (blue dotted line)

Threshold for mania cases (red dotted line)
